# Preparation, biological characterization and preliminary human imaging studies of ^68^Ga-DOTA-IBA

**DOI:** 10.3389/fonc.2022.1027792

**Published:** 2022-12-14

**Authors:** Yingwei Wang, Qixin Wang, Zan Chen, Jian Yang, Hanxiang Liu, Dengsai Peng, Lei Lei, Lin Liu, Li Wang, Naiguo Xing, Lin Qiu, Yue Feng, Yue Chen

**Affiliations:** ^1^ Department of Nuclear Medicine, The Affiliated Hospital of Southwest Medical University, Luzhou, Sichuan, China; ^2^ Nuclear Medicine and Molecular Imaging Key Laboratory of Sichuan Province, Luzhou, Sichuan, China; ^3^ Nuclear Medicine Institute of Southwest Medical University, Luzhou, Sichuan, China; ^4^ Department of Orthopaedics, The Affiliated Hospital of Southwest Medical University, Luzhou, Sichuan, China

**Keywords:** DOTA-IBA, ^68^ Ga, PET/CT, ^99m^ Tc-MDP, SPECT

## Abstract

**Purpose:**

In this study, DOTA-IBA was radiolabeled with ^68^Ga and we determined the optimum labelling conditions and assessed the biological properties of ^68^Ga-DOTA-IBA. We investigated the biodistribution of ^68^Ga-DOTA-IBA in normal animals and undertook PET/CT imaging in humans. Finally, we explored the feasibility ^68^Ga-DOTA-IBA as a bone imaging agent and demonstrated its potential for the therapeutic release of ^177^Lu/^225^Ac-DOTA-IBA.

**Methods:**

The controlled variables method was used to assess the impact of variables on the radiochemical purity of ^68^Ga-DOTA-IBA. The biological properties of ^68^Ga-DOTA-IBA were investigated.^68^Ga-DOTA-IBA micro-PET/CT imaging was performed on animals. Volunteers were recruited for ^68^Ga-DOTA-IBA imaging and data were compared to ^99m^Tc-MDP imaging studies to calculate the target to non-target ratio (T/NT) of the lesions.

**Results:**

The prepared ^68^Ga-DOTA-IBA had a radiochemical purity of >97% and demonstrated good biological properties with a good safety profile in normal mice. PET/CT imaging of the animals showed rapid blood clearance with high contrast between the bone and stroma. Human imaging showed that ^68^Ga-DOTA-IBA could detect more lesions compared to ^99m^Tc-MDP and had a higher targeted to untargeted ratio.

**Conclusions:**

^68^Ga-DOTA-IBA is an osteophilic radiopharmaceutical that can be synthesized using a simple labelling method. ^68^Ga-DOTA-IBA has high radiochemical purity and is stable *in vitro* stability. It is rapidly cleared from the blood, has low toxicity and has strong targeting to the bone with long retention times. We also found that it is rapidly cleared in non-target tissues and has high contrast on whole-body bone imaging. ^68^Ga-DOTA-IBA PET/CT has potential as a novel bone imaging bone modality in patients with metastatic disease.

## 1 Introduction

Cancer remains a significant threat to human health, particularly advanced diseases that have metastasized to the bones. Prostate, breast, lung, liver and thyroid cancers have a high propensity to metastasize to the bones (1–3). Nuclear imaging techniques are widely used in the diagnosis of bone metastases using agents such as [^18^F] ^18^F-NaF and [^99m^Tc] ^99m^Tc-methylene diphosphonate (MDP). [^18^F] ^18^F-NaF requires an accelerator for generation limiting its use in the clinic. Also, [^99m^Tc] ^99m^Tc-MDP often shows false negatives for lesions with no obvious osteogenic response. The value of bisphosphonates in the diagnosis and treatment of skeletal disorders is well established as they have a strong affinity for bone (4). Gallium-68 [^68^Ga] is used clinically as a positron imaging agent and has several advantages. Specifically, [^68^Ga] can be obtained using a germanium gallium generator (^68^Ge/^68^Ga), it is a β-emitter and has a half-life of 67.71 minutes. ^68^Ga/^177^Lu [Lutetium] is a diagnostic and therapeutic nuclide (5).

Ibandronic (IBA) acid is a third generation bisphosphonate. Studies have reported the labelling of metal ions with IBA acid using complex methods requiring larger amounts of precursors and products that have toxic side effects (6, 7). Previous studies have shown that the bifunctional chelate DOTA can be used for the complexation of metal compounds (5). In this study, we combined DOTA with bisphosphate to form a new compound, DOTA-IBA that was then combined with ^68^Ga to form the novel probe, ^68^Ga-DOTA-IBA. In this paper, we describe the preparation conditions and characterize the biological and imaging properties of ^68^Ga-DOTA-IBA.

## 2 Materials and methods

### 2.1 Materials

^68^Ga solution (0.4 M HCL) was eluted from a ^68^Ge/^68^Ga generator (ITG 101, EckertZiegler, Germany). The molecular design of DOTA-IBA was provided by our laboratory and the drug synthesis was provided by the Shanghai New Drug Development Company. Sodium acetate, thin-layer chromatography silica plates and other reagents were purchased from the Shanghai Maclean Biochemical Company. Animal micro positron emission computed tomography (micro-PET/CT; SIEMENS Inveon TM Siemens, Germany), PET/CT (United Imaging 780 Shanghai United Imaging Medical Technology Co., Ltd.). Other equipment, chemicals and animals used in the experiments were provided by the Sichuan Provincial Key Laboratory of Nuclear Medicine and Molecular Imaging. All studies were approved by the Ethics Committee of Southwest Medical University.

### 2.2 Radiolabeling and quality control

A controlled variables approach was used to investigate the effect of the various factors on the radiochemical purity of the markers. The solution was prepared by mixing a certain amount of DOTA-IBA solution (1mg/1ml), sodium acetate solution and ^68^GaCl_3_ solution in sequence. The pH of the solution was adjusted with 0.1 M hydrochloric acid and 0.25 M sodium acetate. The reaction was performed at a specific temperature for a specific time. The solution was cooled and the pH was adjusted to 4-5 before being sterilized and filtered.

Thin layer chromatography silica plates were used as a support and 0.1 M sodium citrate solution was used as a solvent to unfold the system. Quality control images of ^68^Ga-DOTA-IBA: ^68^Ga-DOTA-IBA at the origin (Rf=0.3-0.4); while free ^68^GaCl_3_ moved ahead of the strip with the solvent (Rf=0.9-1.0).

The optimum reaction parameters were 10 μg (10 μL) of DOTA-IBA, 1 ml of sodium acetate and 4 ml of ^68^GaCl_3_. The radiochemical purity of the prepared ^68^Ga-DOTA-IBA was >97%. The pH was 4-5, the reaction temperature was 95°C and the reaction time was 15 min.

### 2.3 *In vitro* stability

The radiochemical purity of ^68^Ga-DOTA-IBA under optimal labelling conditions was determined by incubation in 0.9% NaCl and fresh human serum at 37°C. Paper chromatography was performed at 15 min, 30 min, 1 h, 2 h and 4 h. The experiments were repeated three times and the results are expressed as mean ± standard deviation.

#### 2.3.1 Plasma protein binding rate

Fresh human plasma (0.1 ml) and freshly prepared 0.5 mCi ^68^Ga-DOTA-IBA were added to the test tubes and incubated at 37°C for 2 hours. A 25% trichloroacetic acid solution (1.0 mL) was then added to the tubes and centrifuged for 5 min. The supernatants were collected and the process was repeated three times. The CPM of the supernatant and the precipitate were measured separately using a gamma counter and the plasma protein binding rate (PPB) of ^68^Ga-DOTA-IBA was calculated according to the formula; PPB= [(A-background radioactive count)/(A+B-background radioactive count×2)] × 100%. All results are expressed as the mean ± standard deviation.

#### 2.3.2 Lipid and water distribution

Freshly prepared 0.5 mCi ^68^Ga-DOTA-IBA was added to the tubes before shaking in a vortex mixer for 20 minutes followed by centrifugation for 5 minutes. The upper liquid (organic phase) and the lower liquid (aqueous phase) were collected separately in test tubes. The radioactivity counts of the organic and aqueous phases were measured separately using a gamma counter. The formula lipid-water distribution coefficients (logP) were calculated according to the formula; LogP=log [(B-background radioactive count)/(C-background radioactive count)]. The results were expressed as mean ± standard deviation.

### 2.4 Mouse toxicity tests

24 mice were randomly divided into four experimental groups consisting of a control group and ^68^Ga-DOTA-IBA groups at low, medium and high doses. Each group had equal numbers of male and female animals. The control group was injected with 0.2 ml of 0.9% NaCl and the experimental groups were injected with 0.1 mCi, 0.5 mCi and 1.0 mCi of ^68^Ga-DOTA-IBA solution, respectively. The body weights and general conditions of the mice were observed for 4 weeks. Routine blood tests and liver and kidney function were performed after 2 and 4 weeks in each group. After the 4th week of observation, tissues and organs were harvested from the mice for pathological examination (Ethics committee approval No: KY2022114).

#### 2.4.1 Mouse biodistribution studies

30 healthy mice were divided into 5 groups. All mice were injected with ^68^Ga-DOTA-IBA 0.1 mCi (approximately 0.2ml) *via* the tail vein. Mice were executed under anesthesia at 15 min, 30 min, 1 h, 2 h and 4 h after dosing. Blood, heart, liver, spleen, lung, kidney, stomach, small intestine, brain, femur and muscle tissues were sampled and the radioactivity counts of the different tissues were measured using a gamma counter. The counts were corrected for attenuation and the %ID/g was calculated for each time point. The results were expressed as the mean ± standard deviation.

### 2.5 ^68^Ga-DOTA-IBA imaging analysis in a New Zealand rabbit model

Anesthetized normal New Zealand rabbits were injected with 1.0-2.0 mCi (0.5-0.6 ml) of ^68^Ga-DOTA-IBA under optimal labelling conditions from a marginal ear vein. Whole-body static imaging was performed at 1 h and 3 h after injection using a United Imaging 780 PET/CT instrument. PET images were acquired with a time window of 3.48 ns, an energy range of 350-650 KeV, a 128×128 matrix and a 10 min acquisition time. CT scans were obtained using a tube voltage of 80 Kv, a tube current of 500 Ua and a scan time of 10 min.

### 2.6 ^68^Ga-DOTA-IBA imaging study in normal mice

Whole-body bone imaging using micro-PET/CT was performed at 30 mins, 1.5 h and 3 h after tail vein injection of ^68^Ga-DOTA-IBA 0.2 mCi in anaesthetized mice. The PET imaging parameters were as follows; a time window of 3.48 ns, energy range 350-650 KeV, matrix 128×128, acquisition 10 min. CT scans were obtained at a tube voltage of 80 Kv, and a tube current of 500 As with a scan time of 10 mins.

### 2.7 Establishment of a bone metastasis model and imaging studies

A bone metastasis model was established in nude mice by intertibial bone marrow injection. 25 μL of PC-3 prostate cancer cell culture medium was injected into the left tibia of healthy nude mice. 3-4 weeks after inoculation, the mice were scanned by micro-CT (SIEMENS InveonTM, Munich, Germany) to determine the condition of the bone. The appearance of bone destruction (osteolytic, osteogenic or mixed) in the left tibia indicated that the model was successful. The mouse was anaesthetized with gas anaesthesia (isoflurane) and ^68^Ga-DOTA-IBA 0.1 mCi (50 μl) was injected into the tail vein with an insulin needle under optimal labelling conditions. Whole-body bone imaging was performed 1 h after the injection. Micro-PET/CT and PET acquisitions were performed as described above for normal mice.

### 2.8 ^68^Ga-DOTA-IBA imaging in patients

#### 2.8.1 Patient selection

^68^Ga-DOTA-IBA and ^99m^Tc-MDP whole-body bone imaging were performed in 5 patients and the imaging agents were compared. The patients included 2 males and 3 females who had a mean age of 54.2 years (34-66 years). The interval between the two examinations was > 3 days < 7 days.

The inclusion criteria were patients who had already undergone ^99m^Tc-MDP imaging, had no previous bisphosphonate treatment 1 month before the examination and were able to cooperate well during the examination. The exclusion criteria were patients who has used bisphosphonates within the past month, inability to cooperate during the test and patients who were breastfeeding or pregnant.

All of the above patients were recruited under written consent. This study was approved by the hospital ethics committee and was conducted in compliance with the Helsinki Declaration. Before the ^68^Ga-DOTA-IBA PET/CT examination, patients had routine laboratory blood tests including routine blood counts, and liver and kidney function tests at 7 and 15 days to determine the impact of ^68^Ga-DOTA-IBA on biochemical parameters (Clinical Ethics Registration Number: ChiCTR2200064487).

#### 2.8.2 ^68^Ga-DOTA-IBA PET/CT imaging

The patients were weighed before the examination. An intravenous injection of ^68^Ga-DOTA-IBA with a radiochemical purity of >97% at 0.1 mCi per kg body weight was given. Prior to injection, patients were advised to drink plenty of water and instructed to empty their bladder before imaging. Images were acquired at a tube voltage of 120 Kv, a tube current of 100 mAs and a layer thickness of 5.0 mm. The scanning ranged from the top of the head to the palms of both feet (whole body scan) and was acquired with the patients in a supine position. The foot advanced scanning mode involved a total acquisition of 10-11 beds at 120 s/bed with a subset number 33, an iteration number 3 and a 512×512 matrix. When the reconstruction was complete, the images were processed using PET/CT post-processing software.

#### 2.8.3 Analysis of ^68^Ga-DOTA-IBA and ^99m^Tc-MDP images

^68^Ga-DOTA-IBA and ^99m^Tc-MDP images were analyzed independently in a double-blind manner by two nuclear medicine physicians with > 5 years of experience in diagnostic imaging. Based on the results of the ^68^Ga-DOTA-IBA and ^99m^Tc-MDP imaging agents, the lesions were classified as osteolytic, osteogenic, mixed and normal bone. A lesion was defined as a region of abnormal radioactivity when the uptake value was > the surrounding normal bone background.

### 2.9 Statistical analysis

Statistical analysis was performed using SPSS 26.0. All quantitative information was expressed as the mean ± standard deviation. In the toxicity test, changes in the body weights of each group of mice were compared using repeated measures ANOVA. A P-value threshold of 0.05 was set to indicate statistical significance.

## 3 Results

### 3.1 Radiolabeling and quality control

**Figure 1:**
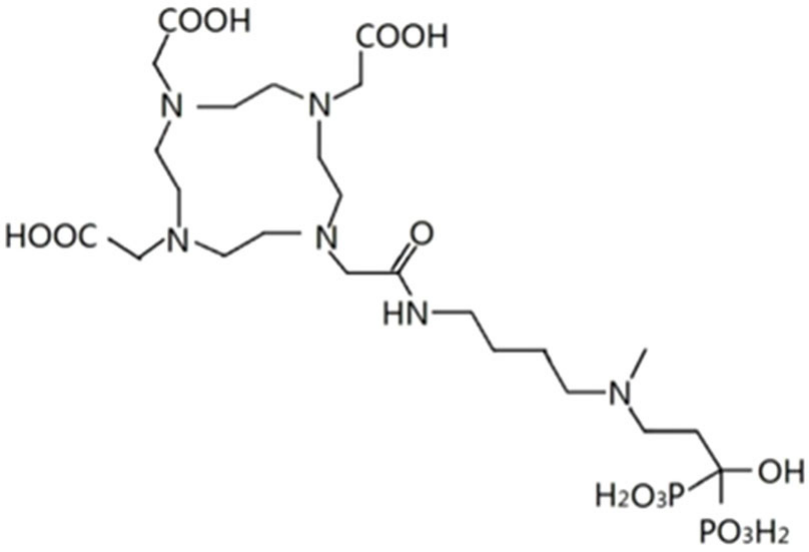
The chemical structure of the DOTA-IBA.

^68^Ga-DOTA-IBA is a third generation bisphosphonate derivative that targets bone metastases. The structure of the ^68^Ga-DOTA-IBA is shown in **Figure 1**. The optimum preparation conditions were 10 μg (10 μL) of DOTA-IBA, 1 ml of sodium acetate solution and 4 ml of ^68^GaCl_3_ drench solution. The radiochemical purity of the prepared ^68^Ga-DOTA-IBA was >97%. The pH of the reaction was 4-5, the reaction temperature was 95°C and the reaction time was 15 min. Thin layer chromatography silica plates were used as a support and 0.1 M sodium citrate solution was used as a solvent to unfold the system. Quality control images of ^68^Ga-DOTA-IBA, ^68^Ga-DOTA-IBA at the origin (Rf=0.3-0.4), while the free ^68^GaCl_3_ moved in front of the strip with the solvent (Rf=0.9-1.0).

### 3.2 *In vitro* stability of ^68^Ga-DOTA-IBA

The *in vitro* stability data for ^68^Ga-DOTA-IBA under different conditions are summarized in **Table 1**. ^68^Ga-DOTA-IBA had good *in vitro* stability at room temperature (26 ± 2°C).

**Table 1:** *In vitro* stability of ^68^Ga-DOTA-IBA under different conditions.

	15 min	30 min	1 h	2 h	4 h
Sanitary saline Room Temp	99.66 ± 0.25%	98.55 ± 0.37%	97.93 ± 0.47%	96.62 ± 0.33%	94.55 ± 0.45%
Blood serum 37°C	99.54 ± 0.33%	98.32 ± 0.32%	96.64 ± 0.33%	94.64 ± 0.35%	91.64 ± 0.33%

### 3.3 Plasma protein binding of ^68^Ga-DOTA-IBA

The PPB of freshly prepared ^68^Ga-DOTA-IBA under optimal labelling conditions was 80.8 ± 0.61% after 1 h incubation in plasma.

### 3.4 ^68^Ga-DOTA-IBA lipid water distribution coefficient

The logP of the lipid-water distribution coefficient of freshly prepared ^68^Ga-DOTA-IBA using the optimal labelling conditions was -2.26 ± 0.03. These data indicated that ^68^Ga-DOTA-IBA has a higher level of water solubility and is less lipid soluble.

### 3.5 ^68^Ga-DOTA-IBA animal toxicity test study

**Figure 2:**
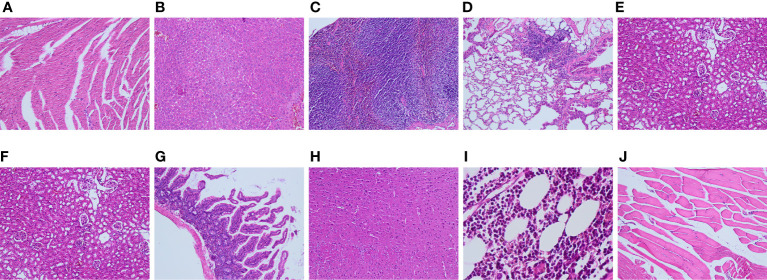
Tissue and organ pathology of mice injected with 1.0 mCi of ^68^Ga-DOTA-IBA (high dose group) for 4 weeks at different magnifications [**(A)**: heart; **(B)**: liver; **(C)**: spleen; **(D)**: lung; **(E)**: kidney; **(F)**: stomach; **(G)**: intestine; **(H)**: brain; **(I)**: bone marrow; **(J)**: muscle)].

All groups of mice in the toxicity test groups showed no abnormalities in the basic health condition at 4 weeks after injection of ^68^Ga-DOTA-IBA. No significant differences in mouse weights were observed between the four experimental groups (P>0.05). At the end of the observation period, samples were isolated from the mice for pathological analysis. The data were discussed with a pathologist and no significant differences in the number, size, morphology and proportion of cells in the tissues of the mice in the high, medium and low dose groups were observed compared to the saline control (**Figure 2** and **Supplement Figure 1–3** HE).

### 3.6 *In vivo* distribution of ^68^Ga-DOTA-IBA in mice

The results of the *in vivo* distribution of ^68^Ga-DOTA-IBA studies in mice are summarized in **Table 2**. From **Table 2**, the blood clearance of ^68^Ga-DOTA-IBA was rapid with only 0.325 ± 0.103% ID/g blood retention at 4 h. Bone had a higher uptake of ^68^Ga-DOTA-IBA that reached a maximum after 2 h (8.365 ± 1.849% ID/g). Uptake was also high in the kidney as ^68^Ga-DOTA-IBA is mainly excreted through the urinary tract.

**Table 2:** *In vivo* distribution of ^68^Ga-DOTA-IBA in mice from 15 min – 4 h (n = 4).

Tissue or organ	%ID/g				
	15 min	30 min	1 h	2 h	4 h
Heart	1.037 ± 0.323	0.557 ± 0.061	0.349 ± 0.051	0.216 ± 0.026	0.159 ± 0.035
Liver	0.526 ± 1.02	0.658 ± 0.212	0.498 ± 0.026	0.418 ± 0.023	0.325 ± 0.041
Spleen	0.665 ± 0.089	0.562 ± 0.026	0.332 ± 0.921	0.350 ± 0.102	0.283 ± 0.052
Lung	0.869 ± 0.152	1.479 ± 0.563	0.659 ± 0.021	0.477 ± 0.141	0.272 ± 0.054
Kidney	4.658 ± 1.263	5.391 ± 1.325	3.774 ± 0.956	2.414 ± 0.145	1.023 ± 0.014
Stomach	1.254 ± 0.251	1.365 ± 0.335	0.964 ± 0.254	0.658 ± 0.214	0.562 ± 0.569
Blood	2.635 ± 0.859	2.837 ± 1.026	1.622 ± 0.562	0.452 ± 0.241	0.325 ± 0.103
Brain	0.097 ± 0.012	0.063 ± 0.011	0.036 ± 0.002	0.039 ± 0.024	0.061 ± 0.045
Femoral	2.318 ± 1.26	3.346 ± 1.32	6.693 ± 1.59	8.365 ± 1.849	4.268 ± 1.231
Muscle	0.472 ± 0.236	0.526 ± 0.548	0.365 ± 0.214	0.194 ± 0.250	0.856 ± 0.114

Percent injected dose rates per gram of tissue are expressed as the mean ± standard deviation.

### 3.7 Imaging analysis of ^68^Ga-DOTA-IBA in New Zealand rabbits

**Figure 3:**
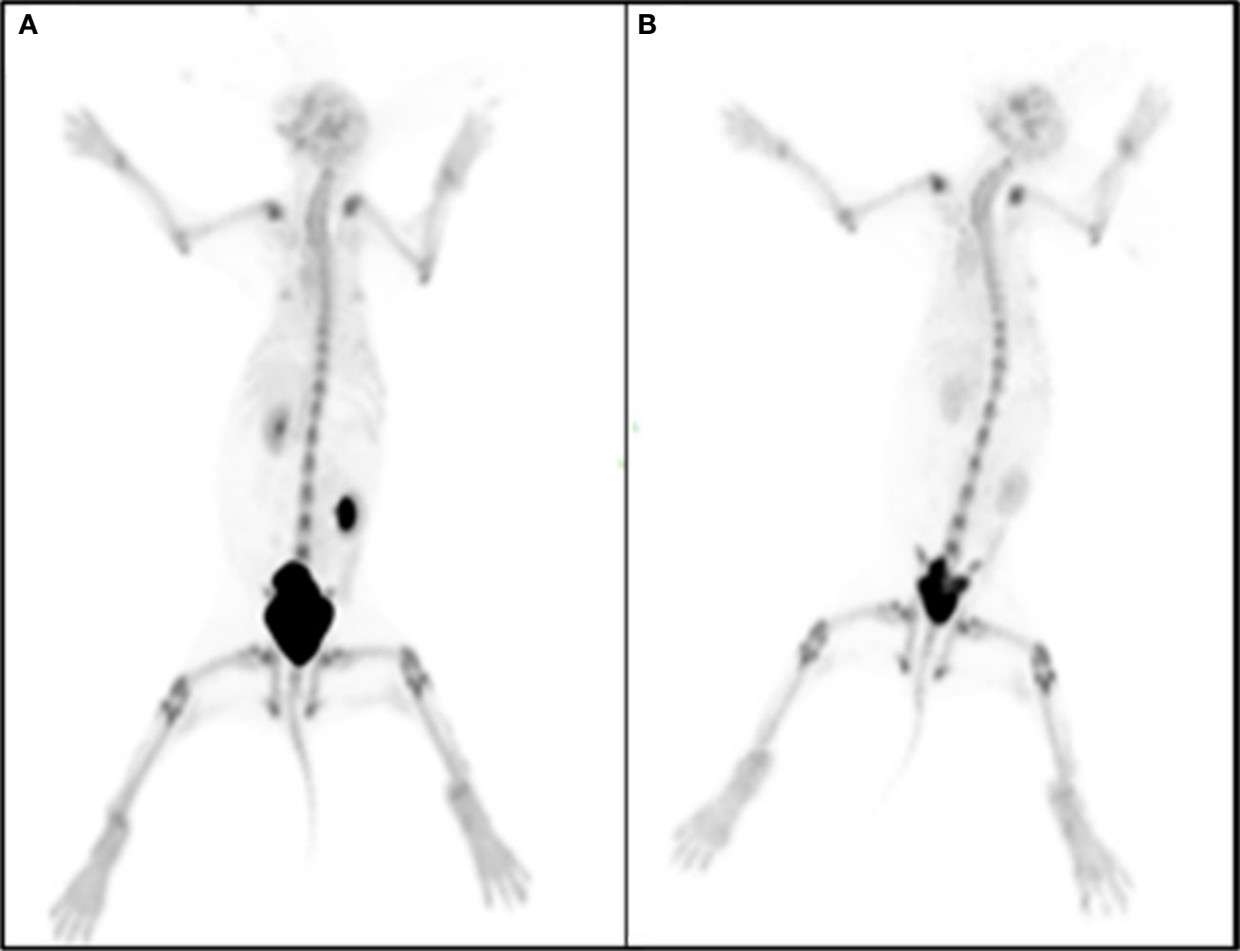
New Zealand rabbits were injected with ^68^Ga-DOTA-IBA 2.0 mCi at 1 h **(A)** and 3 h **(B)** after whole body bone visualization.

Whole-body static bone imaging was performed in New Zealand rabbits at 1 and 3h after intravenous injection of freshly prepared ^68^Ga-DOTA-IBA 2.0 mCi under optimal labelling conditions *via* the ear margins as shown in **Figure 3**. 1 h after injection of ^68^Ga-DOTA-IBA, the rabbit’s urinary tract was clearly visualized and the whole body bone could be seen clearly with the whole spine and limb joints being the most visible. At 3 h after injection, the whole body bone remained clearly visible. In summary, ^68^Ga-DOTA-IBA is a radiopharmaceutical that is excreted through the kidney, and has rapid soft tissue clearance and high skeletal uptake with a long retention time in bone lesions.

### 3.8 Imaging study of ^68^Ga-DOTA-IBA in mice

**Figure 4:**
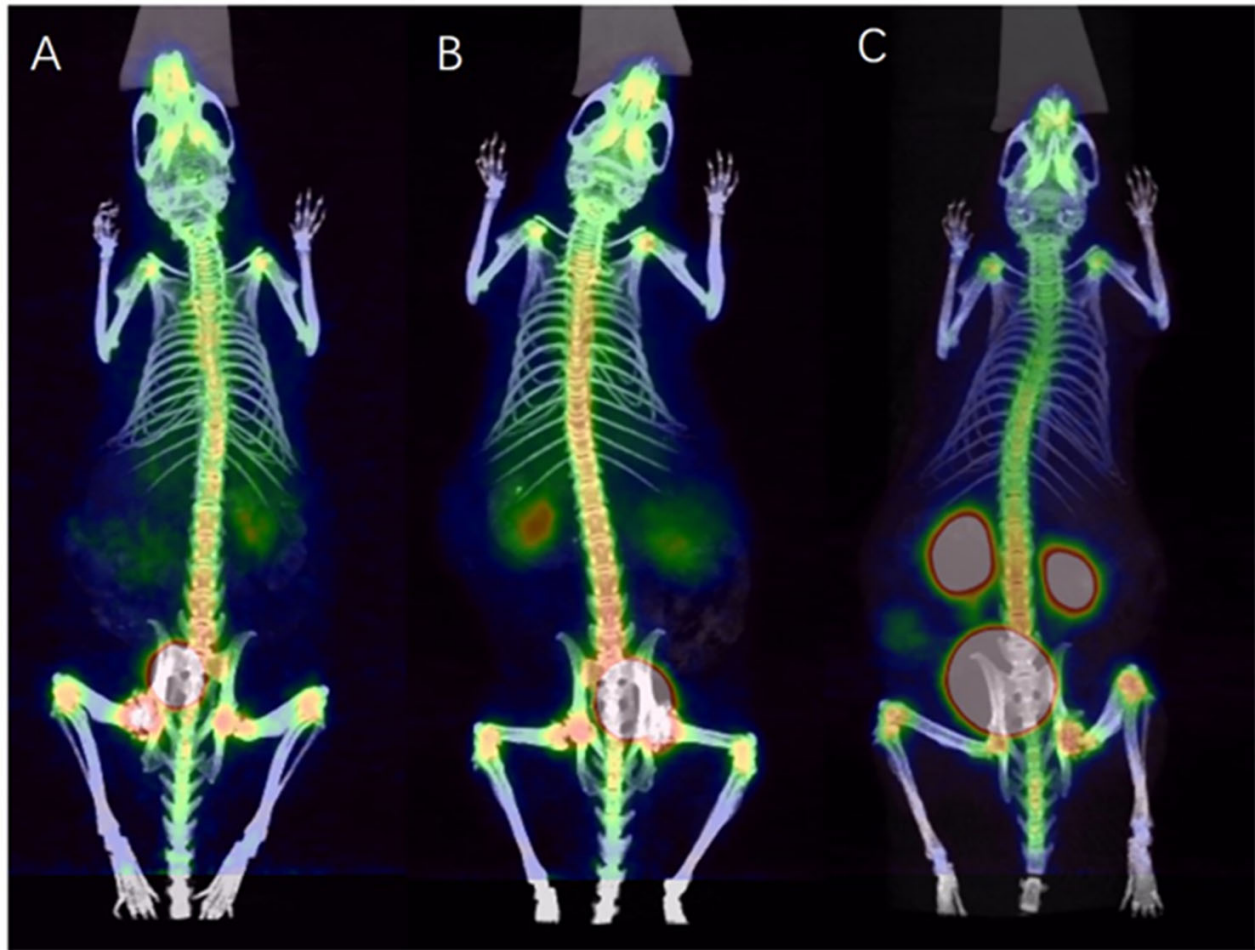
PET/CT images of ^68^Ga-DOTA-IBA in normal mice at different time points [**(A)**: 30 min; **(B)**:1.5 h; **(C)**:3 h].

Whole-body bone images acquired at 30 min, 1.5 h and 3 h after tail vein injection of ^68^Ga-DOTA-IBA 0.2 mCi in mice are shown in **Figure 4**.

### 3.8 Imaging analysis of ^68^Ga-DOTA-IBA in a BALB/c nude mouse model

**Figure 5:**
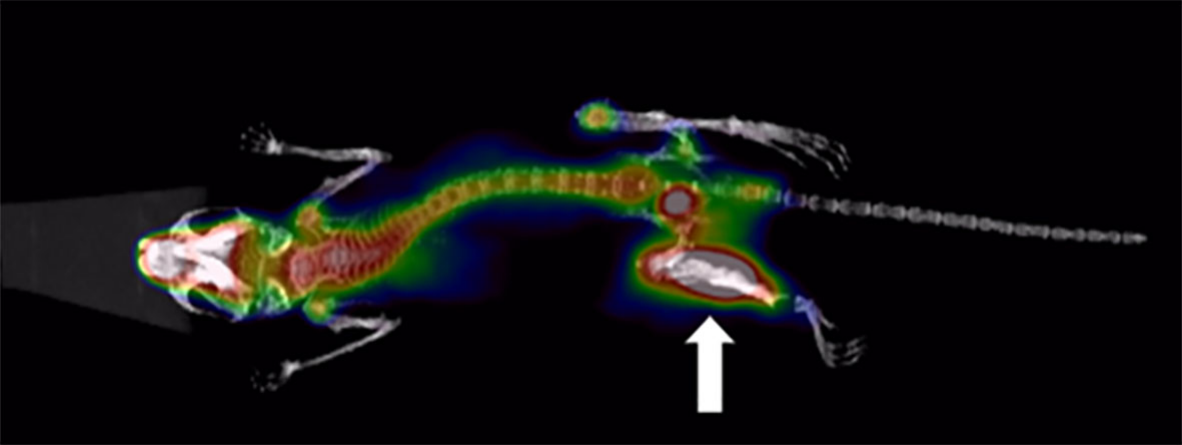
BALB/c nude mice (PC-3) after injection of 0.2 mCi ^68^Ga- DOTA-IBA PET/CT imaging (1.5h).

A whole-body bone image of a BALB/c nude mouse (PC-3) injected with ^68^Ga-DOTA-IBA 0.2 mCi from the tail vein at 1.5 h is shown in **Figure 5**. The model rat showed significant bone destruction in the left tibia with high developer uptake (the arrow) and a SUVmax of 10.3. The T/NT ratio was 6.3 for the lesion.

### 3.10 Preliminary imaging study of ^68^Ga-DOTA-IBA

A total of 5 patients were recruited to the study that consisted of two males and three females who were 34-66 years old with a mean age of 54.2 years. The patient information is summarized in **Table 3**.

**Table 3:** Summary of the basic patient information and clinical diagnosis for ^99m^Tc-MDP imaging results with ^68^Ga-DOTA-IBA.

Number	Gender	Age	^99m^Tc-MDP	^68^Ga-DOTA-IBA	Final diagnosis
1	Female	53	–	–	Breast cancer
2	Female	60	+	+	Breast cancer with bone metastases
3	Male	34	–	–	Lung Cancer
4	Male	58	+	+	Prostate cancer
5	Female	66	+	+	Breast cancer with bone metastases

**Figure 6:**
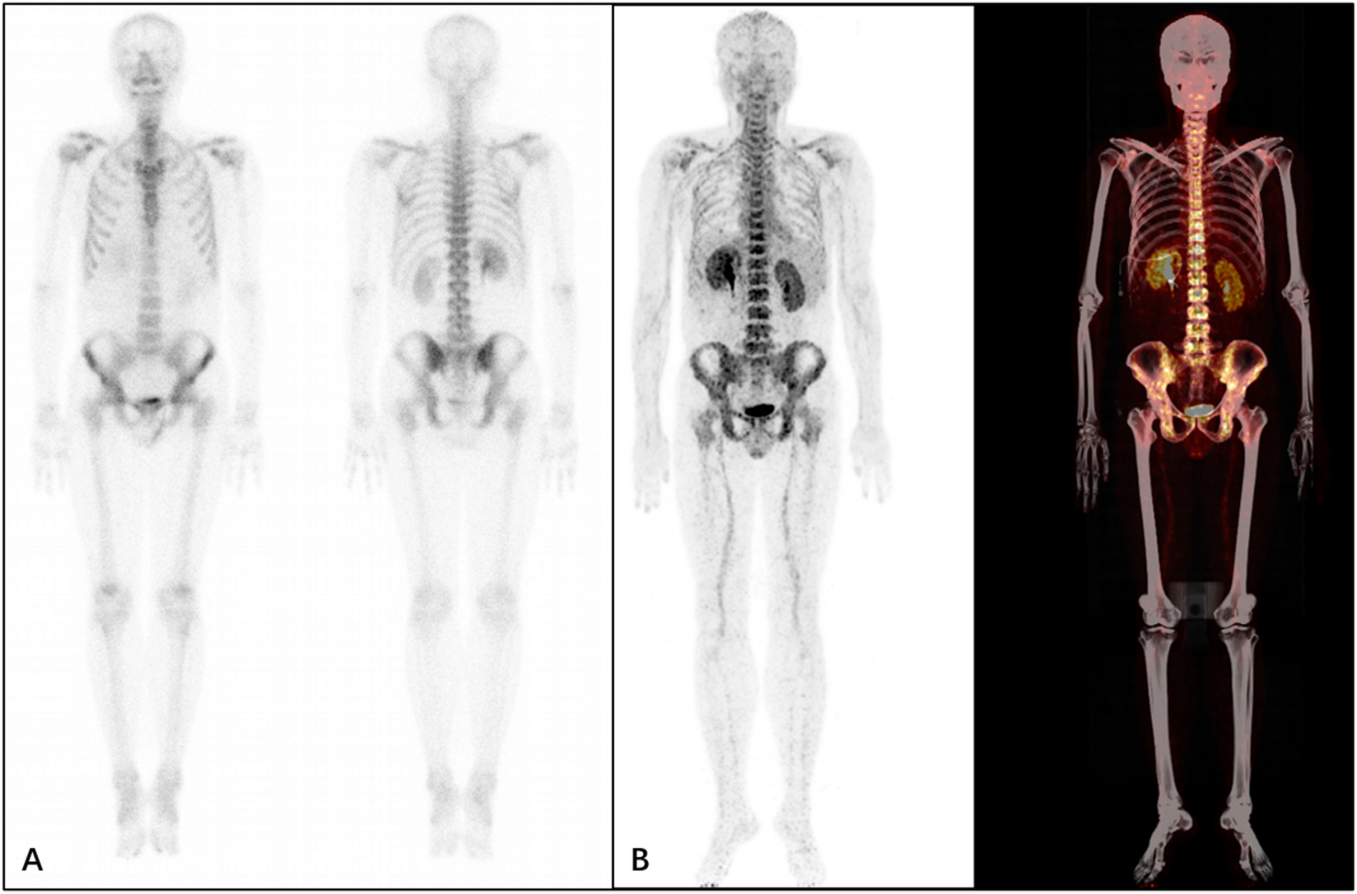
A 34-year-old male patient who underwent resection of a left lung tumor that was discovered 6 months previously. The patient underwent ^99m^Tc-MDP **(A)** and^68^Ga-DOTA-IBA **(B)** imaging. It was observed from the MIP image that the patient’s whole body bones are clear with no abnormal concentrations of developer in the whole body bones.

**Figure 7:**
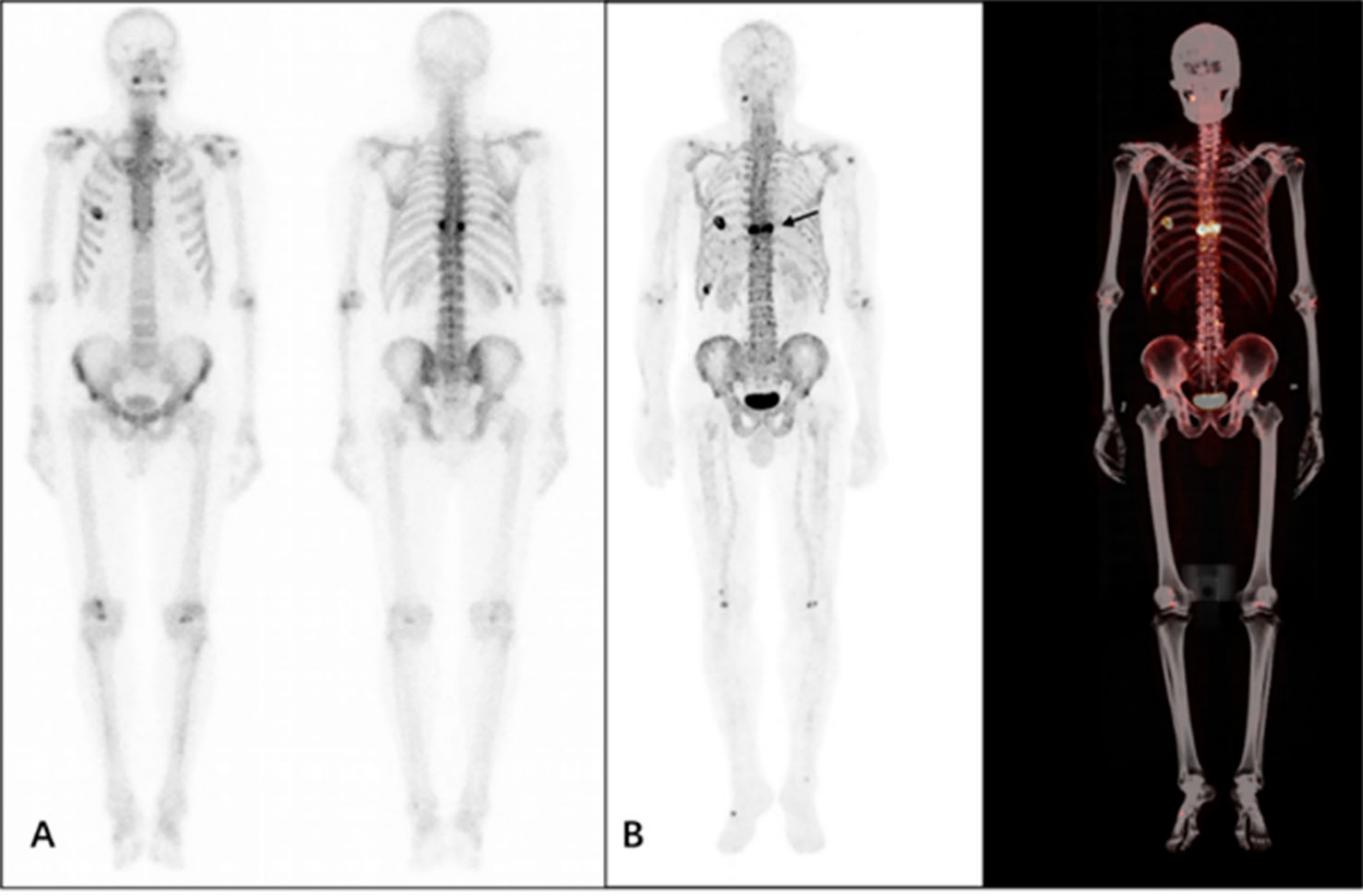
A 58-year-old male patient who underwent surgery for prostate cancer 2 months previously. The patient underwent ^99m^Tc-MDP **(A)** and ^68^Ga-DOTA-IBA **(B)** imaging. From the MIP image, multiple foci of abnormal developer concentrations are visible in the thoracic spine and rib cage. The SUVmax of the lesion shown is approximately 7.8, T/NT 5.8 (arrow).

**Figure 8:**
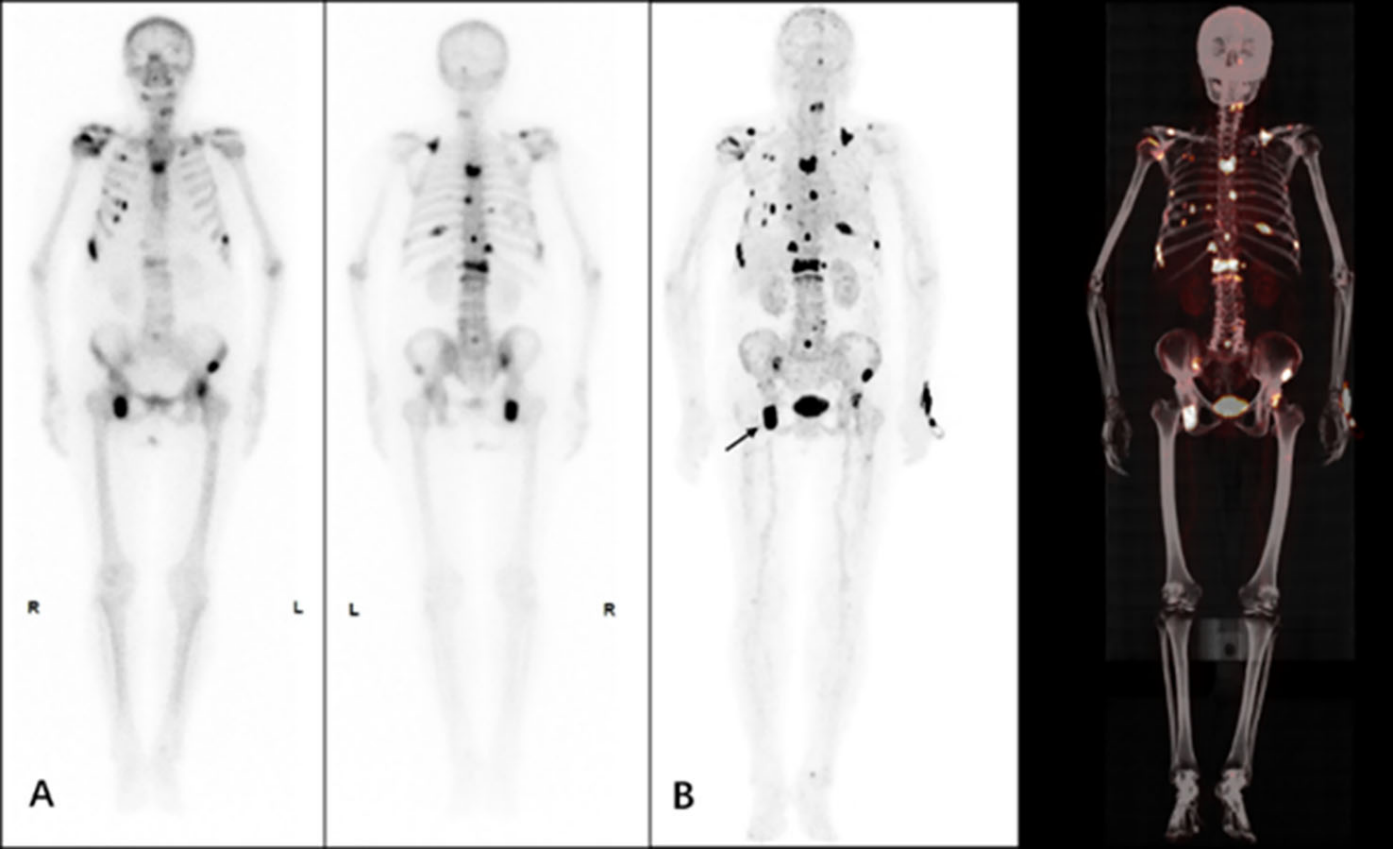
A 60-year-old female patient, who underwent surgery for left breast cancer 3 years previously who had recent generalized skeletal pain. The patient underwent ^99m^Tc-MDP **(A)** and ^68^Ga-DOTA-IBA **(B)** imaging. From the MIP image multiple bone lesions were detected throughout the body with multiple foci of abnormal developer concentrations and a SUVmax of approximately 10.2 for the lesion, T/NT 9.1 (arrow).

**Figure 9:**
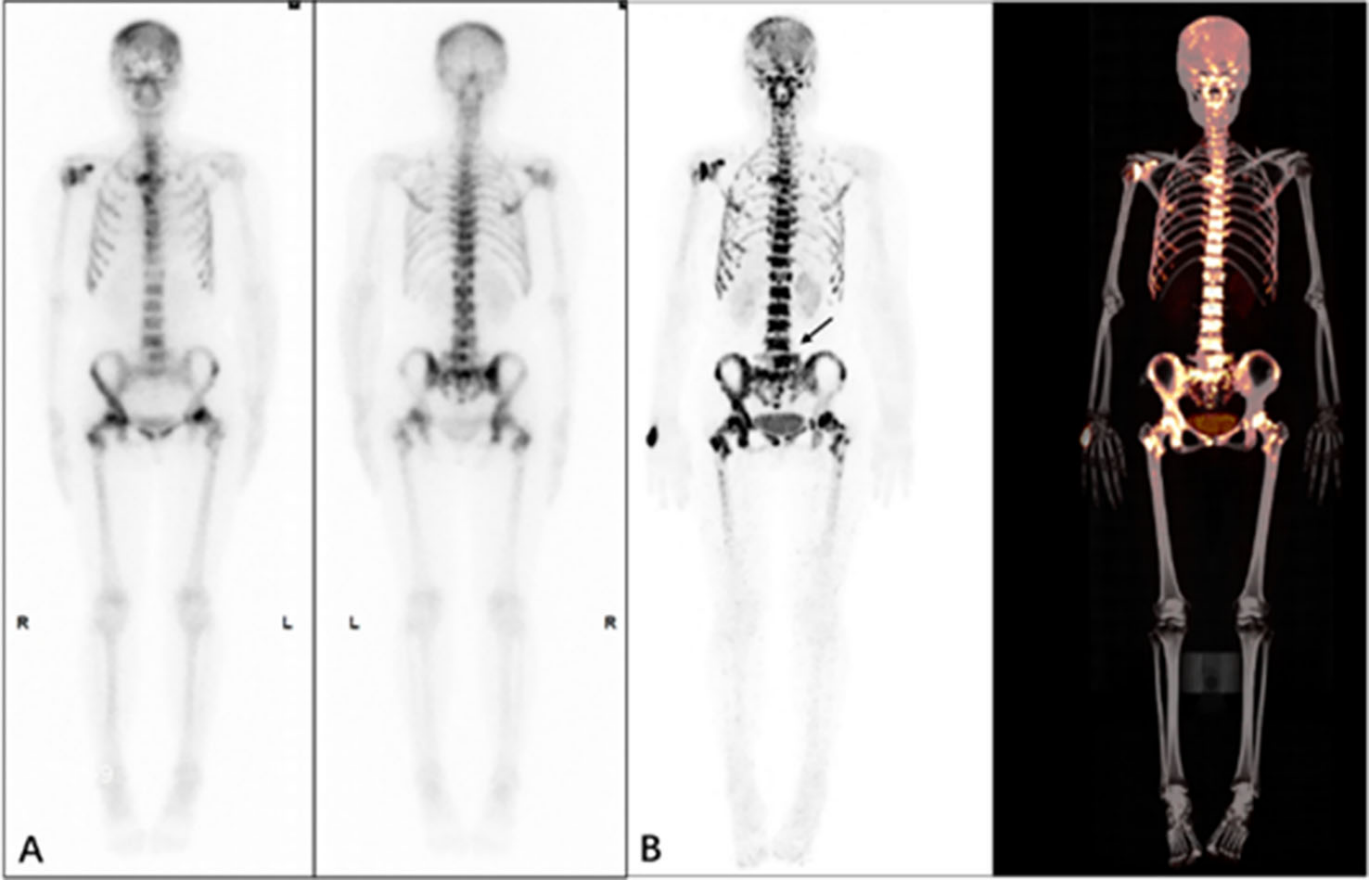
A 66-year-old female patient who underwent surgery for left breast cancer one year ago and now has generalized skeletal pain. The patient underwent ^99m^Tc-MDP **(A)** and ^68^Ga-DOTA-IBA **(B)** imaging. From the MIP image, multiple foci of abnormal developer concentrations can be seen throughout the mesial bones and pelvic bones with a SUVmax of approximately 6.8 for the lesion, T/NT 5.2 (arrow).

From the presented images, ^68^Ga-DOTA-IBA was comparable to ^99m^Tc-MDP in the display of skeletal lesions. ^68^Ga-DOTA-IBA was more sensitive at showing small lesions compared to ^99m^Tc-MDP. The targeted to non-targeted ratio (T/NT) was calculated for ^99m^Tc-MDP SPETCT/CT as 3.2-5.3 and 5.8-9.1 for ^68^Ga-DOTA-IBA PET/CT indicating that ^68^Ga-DOTA-IBA PET/CT lesions had a high targeted to non-targeted (T/NT) ratio (**Figures 6**–**9**).

## 4 Discussion

The clinical use of radiopharmaceuticals has led to improvements in the diagnosis and treatment of cancer and cardiovascular and cerebrovascular diseases (8–12). The continued development of novel radionuclides and molecular probes has led to their increased use in therapeutic radiology applications (13–17). Radionuclide bone imaging is currently the most commonly used method to evaluate abnormalities in bone metabolism. ^99m^Tc-MDP and ^18^F-NaF are the most commonly used bone imaging agents in the diagnosis of bone metastases (8, 18–20). The amount of precursors used in the synthesis of radiopharmaceuticals is critically important in achieving high purity using the smallest amount of precursors for labelling. The DOTA-IBA used in this study is a new precursor and the addition of DOTA allowed the precursor dosage to be reduced to microgram levels. This can significantly reduce the side effects of bisphosphonates for the benefit of patients. Our results show that the radiochemical purity of the ^68^Ga-DOTA-IBA marker was >97% for the specific labelling conditions used. These findings demonstrate the high stability of the compound and its utility as an effective radiopharmaceutical.

Our *in vitro* data showed that ^68^Ga-DOTA-IBA is stable at room temperature (26 ± 2°C) and in saline after preparation and that it remains radiochemically pure at > 97% after 4 h. ^68^Ga-DOTA-IBA was less stable at room temperature (26 ± 2°C), in saline at 37°C and in serum but the overall stability was fair. The results of the plasma protein binding rate showed that ^68^Ga-DOTA-IBA had a higher plasma protein binding rate compared to ^99m^Tc-MDP which has also been observed in other studies (6). These observations may be related to the large molecular weight of DOTA-IBA with a large MDP that can more effectively bind to plasma proteins.

The safety of radiopharmaceuticals is critically important and the aim is to minimize the required dose to maximize imaging or therapeutic efficacy. We showed that after the administration of different doses of ^68^Ga-DOTA-IBA, the mice were not significantly abnormal compared to animals in the control group. These data indicate that ^68^Ga-DOTA-IBA has low toxicity and a strong safety profile supporting its use for subsequent labelling of therapeutic nucleophiles. From the *in vivo* distribution of ^68^Ga-DOTA-IBA in mice, it can be seen that ^68^Ga-DOTA-IBA shows rapid blood clearance because ^68^Ga-DOTA-IBA is mostly excreted through the urinary tract, so the uptake by the kidneys is relatively high (this is related to the kidneys as the main excretory organ). We observed that bone has a high uptake of ^68^Ga-DOTA-IBA that is maintained for 4 h. It also has a high target to non-target ratio of ^68^Ga-DOTA-IBA suggesting that it has strong bone-targeting properties. The findings are consistent with the previous results of other ^68^Ga–bisphosphonate agents (5). In the mouse model imaging studies, ^68^Ga-DOTA-IBA had a high uptake at the lesion and a high target to non-target ratio.

DOTA-IBA contains DOTA and is convenient, efficient and highly labelled when chelating with metal ions and requires very low amounts of precursors. According to our imaging studies, ^68^Ga-DOTA-IBA was sensitive and had a high target to non-target ratio for bone metastases. If the nuclide is replaced by the therapeutic nuclide ^177^Lu/^225^Ac and labelled as ^177^Lu/^225^Ac -DOTA-IBA, it is expected that ^68^Ga/^177^Lu/^225^Ac-DOTA-IBA could potentially be used for the diagnosis and treatment of bone metastases.

Despite the interesting observations reported in this study, our approach has several limitations that need to be refined in future studies. Although 5 volunteers were recruited for the imaging study, the overall sample size in this study was small and did not allow for accurate diagnostic assessment. The comparative study of ^68^Ga-DOTA-IBA with sodium fluoride was not covered in this study and will be further explored in subsequent studies. Follow-up studies will be performed in larger patient cohorts to validate our findings to facilitate the widespread clinical application of our approach in patients with bone metastases.

## 5 Conclusions

In this study, a novel positron-labelled bisphosphonate radiopharmaceutical, ^68^Ga-DOTA-IBA, was successfully prepared. This new imaging agent is simple to prepare, requires a short reaction time, has a high label yield and is stable *in vitro*. Toxicity results showed that it is safe and non-toxic. ^68^Ga-DOTA-IBA has good bone targeting properties, has a high target to non-target ratio and is rapidly cleared from the body. Preclinical PET/CT images demonstrated that ^68^Ga-DOTA-IBA has a higher bone targeting and a higher target to non-target ratio for bone metastases. ^68^Ga-DOTA-IBA is a bone-friendly positron radiopharmaceutical with excellent properties that can be used for the imaging of bone metastases.

## Data availability statement

The original contributions presented in the study are included in the article/**Supplementary Material**. Further inquiries can be directed to the corresponding authors.

## Ethics statement

This study was performed in line with the principles of the Declaration of Helsinki. Study approval was obtained from the hospital’s ethics committee and conducted between September 2021 and August 2022 (Ethics committee approval No.: KY2022114) and (Clinical Ethics Registration Number: ChiCTR2200064487). The patients/participants provided their written informed consent to participate in this study.

## Author contributions

All authors listed have made a substantial, direct, and intellectual contribution to the work and approved it for publication.
