# Supplementary figures and images for: Preparation, biological characterization and preliminary human imaging studies of 68Ga-DOTA-IBA
Source: Front Oncol. 2022 Dec 14;12:1027792. doi: 10.3389/fonc.2022.1027792 (PMC9795169; doi:10.3389/fonc.2022.1027792)

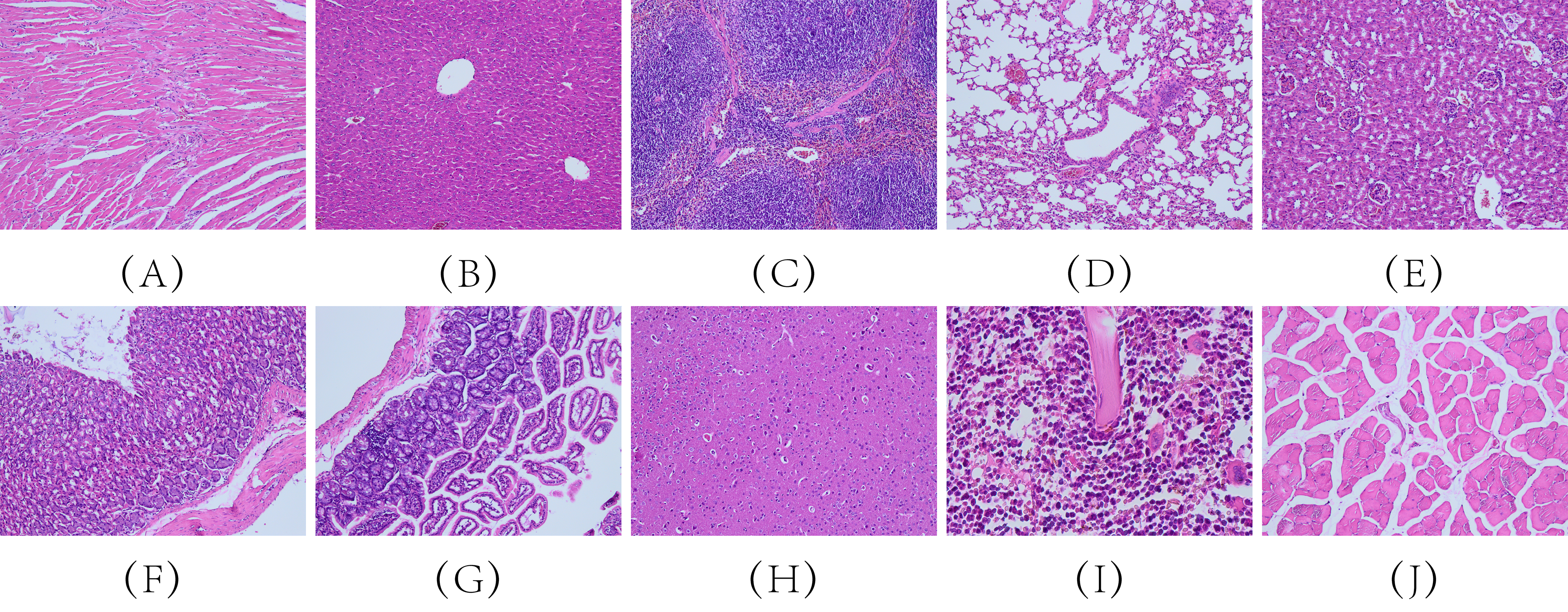

Supplement: Supplementary file 1 [file Image_1.tif]

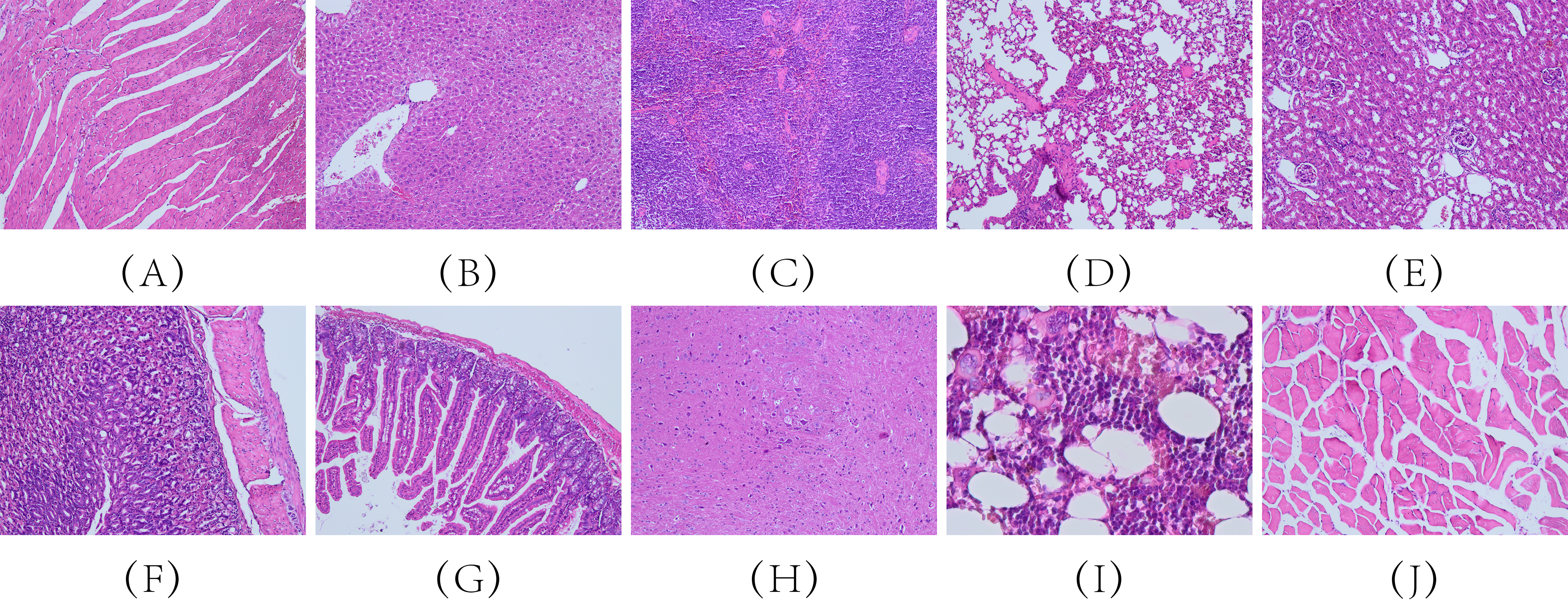

Supplement: Supplementary file 2 [file Image_2.tif]

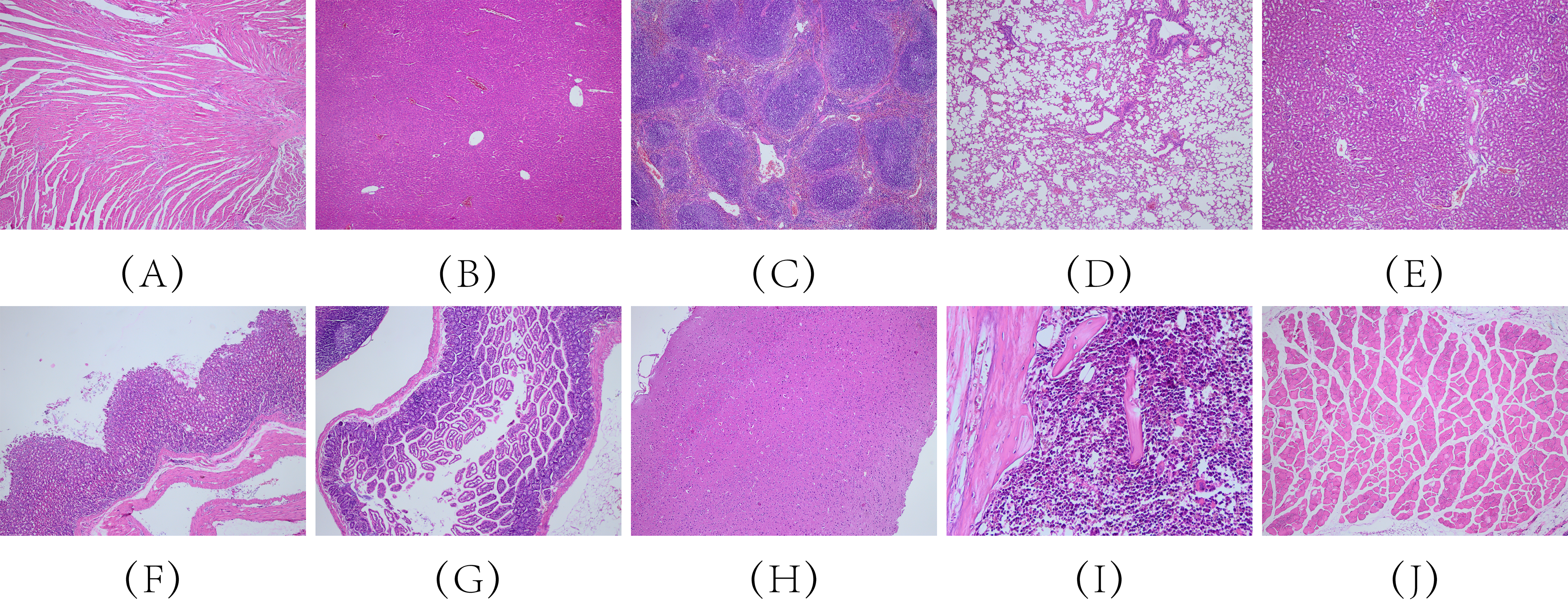

Supplement: Supplementary file 3 [file Image_3.tif]
